# Impact of COVID-19 on football attacking players’ match technical performance: a longitudinal study

**DOI:** 10.1038/s41598-024-56678-y

**Published:** 2024-03-13

**Authors:** Le Luo, Ge Sun, Enkai Guo, Hanbing Xu, Zhaohong Wang

**Affiliations:** 1 College of Humanities and Law, Beijing University of Chemical Technology, Beijing, China; 2 College of Physical Education, Hunan Normal University, Changsha, Hunan China; 3 College of P. E and Sports, Beijing Normal University, Beijing, China

**Keywords:** Public health, Diseases

## Abstract

This study examined the impact of COVID-19 on 28 indicators of match technical performance (MTP) for football attacking players upon their return to play. Analyzing data from 100 players in the Big Five European football leagues, covering 1500 matches each before and after COVID-19 over 3 years (2020–2023), revealed significant differences in 76% of players’ MTP indicators. Notably, 14 indicators, particularly the five indicators linked to scoring, significantly decreased post-COVID-19. On average, players needed 3.09 matches to regain pre-infection MTP levels. The impact varied across player groups, with those in the elite group showing a milder effect; they required an average of 2.64 matches for recovery, compared to the control group’s 3.55 matches. We found that, with increasing age, the majority of players’ MTP indicators did not exhibit significant changes, both before and after they contracted COVID-19. In conclusion, the study highlighted the negative impact of COVID-19 on football attacking players’ MTP. Players in the elite group experienced fewer adverse effects than those in the control group. This insight assisted coaches and managers in evaluating the impact of COVID-19 and similar virus-induced illnesses on players’ MTP, enabling them to formulate training regimens for recovery and specific match tactics upon players' return to play.

## Introduction

The COVID-19 pandemic has significantly impacted football players’ match performance, attracting considerable academic attention. Research is divided into team-level analyses of the pandemic’s effects on football matches and individual-level investigations into players' performance.

Regarding the former, significant studies encompass countries such as Brazil^1^, England^2^, Germany^3^, Italy^4^, Spain^5^, Denmark^6^, and Qatar^7,8^. Additionally, investigations examine changes in home advantage and referee bias when matches are played without spectators^9–11^. Additional studies explore the impact of several performance parameters, including shots, ball possession, and fouls, on match results during the pandemic^12,13^, as well as changes in substitution rules in matches^14^. These findings serve as crucial guidance for teams in adapting their strategies for matches in these challenging times.

Research into the impact of COVID-19 on individual players provides valuable insights for developing specific training and match preparation strategies. Representative studies on players primarily center on the SARS-CoV-2 infection rate^15,16^ and transmission risk after resuming professional football^17–19^, the testing protocol^20–24^ and surveillance for COVID-19^2,25^ and infection control policies for players^26^. Certain studies offer practical suggestions for designing training sessions to aid players in returning to sports following the lockdown imposed by the COVID-19 pandemic^27^. Despite lower exercise intensity in COVID-19-modified training compared to match-play, researchers evaluating the impact of training interventions during home confinement^28^ found that COVID-19-modified training might be safer than small-side game training^29^. Scholars have found that training for strength and conditioning plays a significant role in the training habits formed during COVID-19 confinement^30^. The conclusion drawn is that training programs developed during confinement prioritize physical condition and functional emphasis^31^. Studies indicate that dedicating additional training time to improve jumping, strength, and sprinting abilities, along with implementing other management strategies, can aid female athletes in sustaining higher fitness levels during the lockdown imposed by the pandemic^32^.

Injuries are a crucial aspect when examining how individual players are impacted by the COVID-19 pandemic. Scholars hold varying perspectives on this matter. Two studies indicate that Serie A players faced a heightened occurrence of muscular injuries in the competitive season post the COVID-19 lockdown, compared to the pre-pandemic period^33,34^. A study in a Japanese professional football league revealed a significant increase in the incidence of muscular injuries in the two months after the COVID-19 pandemic lockdown^35^. After the lockdown and the 2020 season restart, match injuries decreased, but the frequency and severity of training injuries increased compared to the period from 2015 to 2019^36^. However, a systematic review found no discernible difference in injuries between pre- and post-COVID-19 breaks in most studies^37^. This standpoint is reinforced by research conducted in La Liga^38^. The explanation for these findings could be attributed to the argument that increasing player substitutions from three to five per match might reduce the external load on players, consequently lowering the risk of injuries during matches^14^.

After contracting COVID-19, football players experienced considerable psychological difficulties, including increased stress, anxiety, depression, and psychological distress^39^. These psychological challenges may increase the players’ demand for cognitive activities during training or matches, subsequently leading to mental fatigue^40^. Scholars have discovered that mental fatigue negatively influences both offensive techniques, such as passing and shooting^41^, and defensive techniques, such as tackling^42^. COVID-19 anxiety and competitive anxiety could hinder athletic performance in pandemic competitions^43^. Nevertheless, when players moved toward fewer restrictions and a regular training situation, this improved, although depression symptoms were prevalent early in the lockdown^44^. The study indicates that high resilience and low trait anxiety are pertinent factors for mental health among elite football players during the pandemic^45^. Additionally, it underscores the importance of maintaining a healthy mental state through regular exercise in a safe home environment and adhering to a consistent physical activity schedule^46^.

It is crucial to note, however, that scientific training courses, injury prevention programs, or psychological interventions cannot directly dictate the results of a match. Match results are indeed contingent on the players' performance during the competition. According to researchers, the COVID-19 lockdown adversely affected the physical performance of professional football players^47,48^. The COVID-19 pandemic was linked to a decrease in physical match performance across La Liga, Serie A, Polish Ekstraklasa, and Croatian HNL, as indicated by the systematic review of eleven articles. Interruptions in match and group training lasting up to 15 weeks and 8 weeks, respectively, seemed to contribute to this effect^37^. The primary indicators of football players’ physical performance, as per the aforementioned study, include average peak velocity, high-intensity running distance, low-intensity running distance, total distance, sprinting distance, and others. Persistent symptoms post-infection, such as general fatigue and muscle weakness, significantly reduce players’ capacity for high-intensity competition^49^. Recovery in aerobic capacity is particularly delayed, affecting players’ game readiness, whereas anaerobic capacity is comparatively stable^50,51^. Chronic health issues like breathlessness, cough, and cardiopulmonary complications present additional obstacles to regaining optimal performance^52^. The observed decline in physical and mental health post-infection underscores the need for customized recovery programs^53^. These findings highlight the essential role of comprehensive health management in restoring players’ competitive capabilities post-COVID-19. Furthermore, the physical fatigue that may arise earlier or more frequently in matches due to the aforementioned conditions can further negatively affect the technical fundamentals of passing, dribbling, and kicking, as research has indicated that technical skills dependent on movement precision and efficiency are adversely impacted by physical fatigue^54^.

The team secures victory in a football match by scoring more goals than its opponent. This is achieved through a variety of match technical performances, such as shooting, dribbling, and passing. Even without considering factors such as COVID-19 infection, evidence suggests that mid-season breaks longer than 13 days may negatively affect momentum and performance.^55^. Arguments presented by Link and Anzer supported the idea that player performance might change or decline following the resumption of football matches during the pandemic. Their study revealed noticeable performance differences in both Bundesliga and Bundesliga 2, with the latter experiencing the most significant changes^11^. Research by Jamil and Kerruish on 637 Premier League players found that younger wingers (16–25) had more shots on target and attempts from open play than their older counterparts. Similarly, strikers aged 21–25 were found to be more effective in scoring and shooting from outside the box compared to those aged 26–30^56^. Fischer et al. examined the pre- and post-COVID-19 match performance of Bundesliga and Serie A players. They observed a more substantial decline in pass performance among players over 30 years old compared to those under 25 and those between 26 and 30 years old^57^. However, the absence of players from the La Liga, and Ligue 1 in the sample meant that many prominent players were not considered in the study.

Savicevic et al. emphasized the importance of understanding the impact of COVID-19 infection on an athlete’s physical capabilities. However, there is limited research on how COVID-19 infection affects an athlete’s working capacities^58^. Moreover, to our knowledge, few studies have investigated how COVID-19 infection influences players’ performance in real sporting environments.

This study aims to evaluate whether football players, after recovering from COVID-19 and returning to play, exhibit significant changes in various indicators of match technical performance (MTP) compared to their pre-infection MTP levels. This will be accomplished by comparing the post-COVID MTP of players who contracted COVID-19 from the Big Five European football leagues (referred to as the Big Five) to their pre-COVID MTP. Due to varied MTP evaluation indicators for football players in different positions, our focus will be on attacking players, as their ability to exploit goal-scoring opportunities and their skill in orchestrating the offense are critical to team success, especially in crucial matches^59,60^. Building on the aforementioned literature on players’ match performance, we present the following hypotheses:

### H1a

 The post-COVID MTP of attacking players decreases compared to their pre-COVID MTP.

### H1b

 Elite attacking players experience a smaller decrease in post-COVID MTP compared to their pre-COVID MTP than ordinary attacking players.

### H2a

 Attacking players need a specific number of matches to regain their pre-COVID MTP level.

### H2b

 Elite attacking players need fewer matches to recover their pre-COVID MTP level compared to ordinary attacking players.

### H3a

 Older attacking players need more matches to recover their pre-COVID MTP level with aging.

### H3b

 Elite older attacking players need fewer matches than ordinary older attacking players to regain their pre-COVID MTP level.

### H4a

 Older attacking players undergo a more substantial decrease in post-COVID MTP compared to pre-COVID MTP.

### H4b

 Elite older attacking players experience a smaller decrease in post-COVID MTP compared to their pre-COVID MTP than ordinary older attacking players.

## Methods

### Data

Data from 100 players’ matches were selected as the sample. First, we identified the date of each player's positive COVID-19 test via official club announcements. In cases where a player tests positive for COVID-19 multiple times, the date of their initial positive diagnosis is recorded. Second, the sample of pre- and post-COVID-19 matches for each player was created by tracking 15 matches both retrospectively and prospectively before and after the confirmed date. Given that domestic professional leagues typically play one match per week, 15 matches typically take place over 3–4 months. This duration surpasses the 60-day measurement of players’ post-COVID aerobic capacity conducted by Parpa and Michaelides^61^ but remains shorter than the multi-year study conducted by Fischer et al.^57^. This timeframe ensures the acquisition of data on the MTP indicators, reflecting players’ relatively stable performance over a given period. Additionally, it minimizes changes in MTP data caused by variations in players' physical fitness due to age. Particularly for older players, when the research period extends to 1–2 years or even longer, avoiding the objective phenomenon of match performance decline caused by natural aging becomes challenging. A study investigating the effects of age on match performance in football players revealed that players, on average, experienced a 0.56% decrease in their total distance covered for each year of aging. Similarly, the number of high-intensity efforts and distance covered by high-intensity running decreased by 1.80% and 1.42% per year, respectively^62^.

Our data collection is exclusively focused on the Big Five domestic leagues to minimize outliers from matches against lower-tier teams (a situation often occurring in cup competitions) and to establish a consistent evaluation framework. By analyzing 15 matches for each player both before and after their COVID-19 infection, we ensure comprehensive coverage of league opponents, thereby capturing a broad spectrum of performance data. This method accurately reflects players' true capabilities amidst variations in team strengths and individual forms, benefiting from the regular league match schedule, which is predominantly set for weekends. In contrast, European and domestic cup competitions, generally held mid-week or occasionally on weekends, and whose schedules may vary based on match outcomes, unpredictably impact players' recovery and performance due to scheduling inconsistencies and each player’s unique calendar. Given the varying times at which each player contracted COVID-19, our research data spans from 2020 to 2023, covering nearly three years. This longitudinal study, extending several years after the pandemic's onset, provides a detailed view of performance metrics that encapsulate league involvement and indirectly reflect the impact of participating in all types of competitions. This approach mirrors the realities of professional footballers’ career dynamics, providing an all-encompassing insight into competitive performance.

While some matches may be duplicated within the total of 3000 matches (for example, Mbappe and Neymar both played for Paris Saint-Germain versus Lens on September 27, 2020), the focus of the study is specifically on individual match data for each player. Consequently, the data for each player are considered independent of one another.

Our study leverages data from FBref.com, powered by Opta Sports, known for its precision and widely used in industries such as betting, media, and professional sports analysis^55,59^. This reliable data supports our football research, contributing to significant academic inquiries^63–67^. All data acquisition and the analysis of match data did not involve any additional testing or human experiments. The entire research was conducted in accordance with relevant guidelines and regulations.

### Player selection

Our study concentrates on players from the Big Five leagues, renowned for their global prestige and ability to attract world-class talent, as they represent the pinnacle of football competition^68,69^. We specifically target attacking players, identified as forwards in the Premier League, La Liga, and Ligue 1, and as strikers in the Bundesliga and Serie A, per the leagues’ official websites.

To conduct a secondary verification of players' positions through third-party channels and to accomplish the task of selecting elite players, we rely on FIFA 23 player ratings, a credible source from the widely recognized football simulation video game licensed by FIFA, covering the period from 1993 to 2023. This game includes over 19,000 players, more than 700 teams, and 30 global events^70^, providing a rich dataset invaluable for coaches and sports analysis^71,72^. The use of this video game data for analytical purposes, including predicting match outcomes and evaluating player wages with machine learning, has become increasingly popular since 2014^73,74^. For example, researchers have utilized it in machine learning projects to accurately predict match results^75^ and determine if a player’s wage is above or below the median based on age and overall attributes^76^. For our analysis, attackers within the top 50 FIFA 23 overall ratings from the Big Five were selected as the elite group. Should any selected player lack confirmed COVID-19 infection data, the next highest-rated attacker serves as a substitute. Conversely, attackers from the Big Five in the bottom 50 of the ratings constitute the control group, with substitution criteria applied equivalently. Ultimately, the distribution of the 100 attacking players across the Big Five is presented in Table 1, closely aligned with the UEFA association rankings.

**Table 1:** Distribution of 100 attacking players in the Big Five European Football Leagues.

2022/23 season Ranking	Country	Points	Top League	Number of players included in the sample
1	England	109.570	Premier League	24
2	Spain	92.998	La Liga	21
3	Germany	82.481	Bundesliga	18
4	Italy	81.926	Serie A	26
5	France	61.164	Ligue 1	11

The association club coefficients are based on the results of each association’s clubs in the five previous UEFA Champions League and UEFA Europa League seasons^92^. If players change the clubs or leagues they play for in the 15 matches before or after contracting COVID-19, we consider the clubs and leagues they are playing for at the time of our statistical analysis.

**Figure 1:**
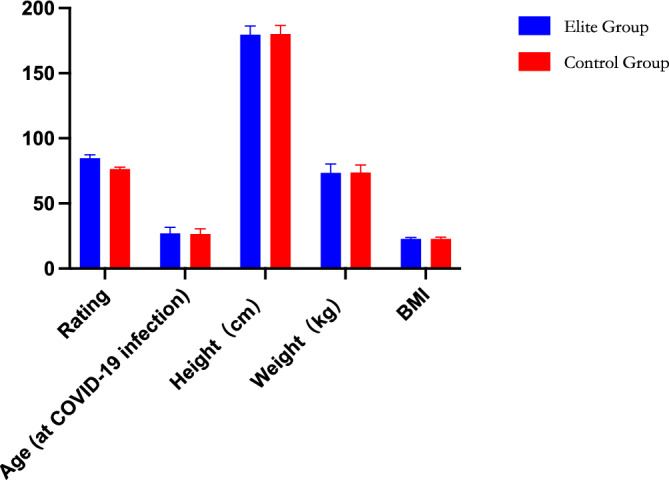
Comparative analysis of fundamental attributes between elite and control groups of 100 attacking players.

In the elite group, Messi boasts the highest rating (91), while Giroud holds the lowest (82), resulting in an average of 84.76. In contrast, within the Control Group, the average score stands at 76.48, with Ruben Garcia securing the highest at 79 and Sulaimana attaining the lowest at 75. A statistically significant difference exists between the two player groups concerning overall ratings (*p* < 0.001) but not in relation to other indicators (Fig. 1).

### Indicator selection

The study uses 28 MTP indicators for attacking players, as defined in Table 2.

**Table 2:** Indicators of MTP for attacking players.

Indicators	Definition	Categories	Subcategories
xG	xG totals include penalty kicks, but do not include penalty shootouts (unless otherwise noted)	Scoring	Expected
npxG	Non-Penalty Expected Goals	Scoring	Expected
xAG	Expected Assisted Goals. xG which follows a pass that assists a shot	Scoring	Expected
SCA	Shot-Creating Actions. The two offensive actions directly leading to a shot, such as passes, take-ons and drawing fouls	Scoring	SCA
GCA	Goal-Creating Actions. The two offensive actions directly leading to a goal, such as passes, take-ons and drawing fouls	Scoring	GCA
ShortCmp	Passes Completed (Short: passes between 5 and 15 yards)	Passing	Short
ShortAtt	Passes Attempted (Short: passes between 5 and 15 yards)	Passing	Short
ShortCmp%	Passes Completion (Short: passes between 5 and 15 yards)	Passing	Short
MedCmp	Passes Completed (Medium: passes between 15 and 30 yards)	Passing	Medium
MedAtt	Passes Attempted (Medium: passes between 15 and 30 yards)	Passing	Medium
MedCmp%	Passes Completion (Medium: passes between 15 and 30 yards)	Passing	Medium
LongCmp	Passes Completed (Long: passes over 30 yards)	Passing	Long
LongAtt	Passes Attempted (Long: passes over 30 yards)	Passing	Long
LongCmp%	Passes Completion (Long: passes over 30 yards)	Passing	Long
Touches	Number of times a player touched the ball Receiving a pass, then dribbling, then sending a pass counts as one touch	Possession	Touches
Def Pen	Touches in defensive penalty area	Possession	Touches
Def 3rd	Touches in defensive 1/3	Possession	Touches
Mid 3rd	Touches in middle 1/3	Possession	Touches
Att 3rd	Touches in attacking 1/3	Possession	Touches
Att Pen	Touches in attacking penalty area	Possession	Touches
TouchesLive	Live-ball Touches. Does not include corner kicks, free kicks, penalties, goal kicks, or throw-ins	Possession	Touches
DriAtt	Take-Ons Attempted. Number of attempts to take on defenders while dribbling	Possession	Take-Ons
DriSucc	Successful Take-Ons. Number of defenders taken on successfully, by dribbling past them. Unsuccessful take-ons include attempts where the dribbler retained possession but was unable to get past the defender	Possession	Take-Ons
DriSucc%	Percentage of Take-Ons Completed	Possession	Take-Ons
DriMis	Mis-controls. Number of times a player failed when attempting to gain control of a ball	Possession	Carries
DriDis	Dis-possessed. Number of times a player loses control of the ball after being tackled by an opposing player. Does not include attempted take-ons	Possession	Carries
ReceivingRec	Passes Received. Number of times a player successfully received a pass	Possession	Receiving
ReceivingPrgR	Progressive Passes Received. Completed passes that move the ball towards the opponent's goal line at least 10 yards from its furthest point in the last six passes, or any completed pass into the penalty area. Excludes passes from the defending 40% of the pitch	Possession	Receiving

The mentioned indicators, algorithms, and player data are sourced from FBref and Opta. The primary focus of this study revolves around three pivotal dimensions of MTP concerning attacking players: possession, passing, and shooting^90^.

### Study design

A series of independent t-tests were conducted to evaluate whether attacking players, post-COVID-19 infection, demonstrated a decline in diverse MTP indicators upon their return to the field compared to their individual pre-COVID-19 MTP data. This methodology was similarly applied to investigate potential distinctions in the negative impact on MTP indicators between the elite and control groups, both having experienced COVID-19 infection.

To assess the recovery progress of attacking players post-COVID-19 contraction, we calculated the average values of each MTP indicator for the 15 matches preceding their infection. Subsequently, we compared these averages with the respective data from matches played by the players upon their return. If, in the Xth match, a player's performance in a specific MTP indicator equaled or surpassed their pre-COVID-19 average for that indicator, it was deemed that the player had reached their pre-COVID-19 level in that particular aspect after X matches. This straightforward assessment method facilitated the evaluation of recovery time for players who had experienced COVID-19 and subsequently resumed playing.

In testing the hypothesis concerning age, linear regression analyses are conducted with age as the independent variable and MTP indicators such as xG as the dependent variable. The objective was to investigate the association between age and the indicators and determine whether older attacking players needed more matches to regain their pre-COVID-19 levels.

According to Hayes, the effect of X on some variable Y is moderated by W if its size, sign, or strength depends on or can be predicted by W. In that case, W is said to be a moderator of X’s effect on Y^77^. In this study, X represents the age of the players at the time of COVID-19 infection. W signifies the pre- or post-COVID-19 status, presented as a binary variable with W = 1 denoting pre-COVID-19 and W = 2 indicating post-COVID-19. The MTP indicators mentioned earlier serve as Y. When a specific MTP indicator is considered Y, the remaining indicators act as control variables, and so forth. This methodology aims to explore whether the impact of player age on each MTP indicator is influenced by COVID-19 infection status.

## Results

### 76% of players showed differences in MTP indicators after COVID-19 infection

**Figure 2:**
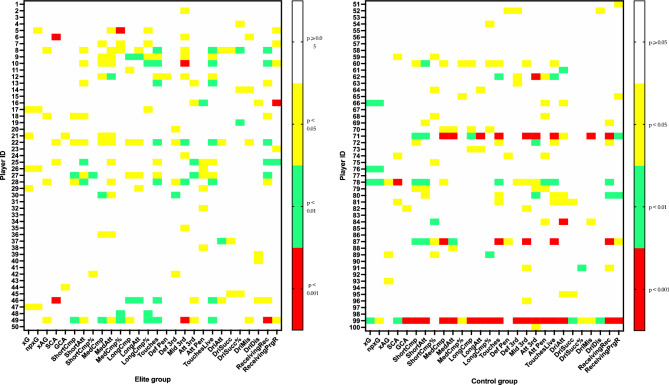
Comparison of significant differences in 28 MTP indicators of 100 attacking players before and after COVID-19 infection (**A**).

As illustrated in Fig. 2, players in both the elite (left) and control (right) groups exhibited numerous indicators that were significantly different before and after contracting COVID-19. We observed that 76% of the 100 attacking players who contracted COVID-19 displayed significant differences in at least one MTP indicator compared to their pre-COVID-19 levels upon returning to the match. Among them, 92% of the elite group players met the criteria, while only 85% of the control group players did. On average, each player had 3.2 MTP indicators showing significant differences after contracting COVID-19. The elite group had an average of 3.4 indicators, while the control group had an average of 3 indicators.

**Figure 3:**
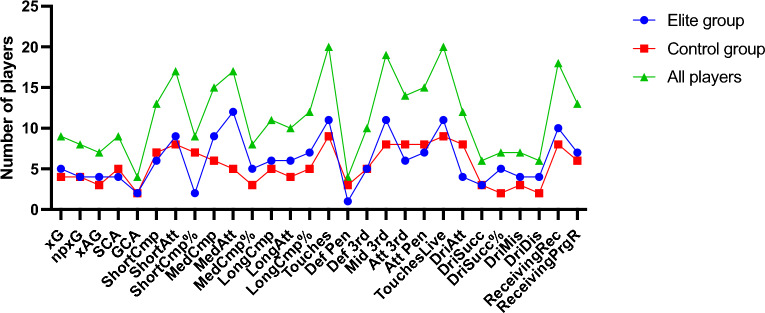
Comparison of significant differences in 28 MTP indicators of 100 attacking players before and after COVID-19 infection (**B**).

The Touches and Touches Live indicators, each with twenty cases, exhibited the most notable changes following COVID-19 infection, as depicted in Fig. 3. In contrast, Def Pen and GCA had only four cases each and demonstrated the least significant differences. Def Pen had the fewest cases in the elite group, with just one, but MedAtt displayed the most, with twelve cases of significant differences. Within the control group, DriSucc% and DriDis had the fewest occurrences—just two each—while Touches and Touches Live had the most significant differences—nine appearances.

### The performance of attacking players was weakened by COVID-19, with the elite group being less affected than the control group

Independent-samples t test was conducted on the MTP indicators of players before and after COVID-19 infection, revealing that the p values of fourteen MTP indicators, including xG and npxG, were less than 0.05, indicating significant differences. The mean values of the aforementioned MTP indicators before COVID-19 infection were all higher than those after COVID-19 infection (Table 3). From the table, we observed significant decreases in xG, npxG, xAG, SCA, and GCA, which are all scoring-related indicators, for attacking players after contracting COVID-19. In the passing category, only LongCmp, involving long passes, showed a significant decrease in attacking players after COVID-19 infection compared to before. Regarding the possession category, the number of touches by attacking players in Mid 3rd, Att 3rd, and Att Pen significantly decreased after COVID-19 infection, particularly in Att 3rd, where the number of touches decreased by 1.057 times per game compared to pre-COVID-19. In the take-ons subcategory, the DriAtt and DriSucc also showed significant declines. After COVID-19 infection, attacking players received 1.527 fewer passes per game compared to pre-COVID-19. These results partially support hypothesis H1a.

**Table 3:** Indicators of significant differences in attacking players pre- and post-COVID-19 infection.

Indicators	Group	N	Mean	SD	t	df	Two-sided p	Mean difference
xG	pre-COVID-19	1500	0.3023	0.38449				
	post-COVID-19	1500	0.2531	0.35932	3.621	2984.359	0	0.04920
npxG	pre-COVID-19	1500	0.258	0.33034				
	post-COVID-19	1500	0.2223	0.30558	3.075	2979.991	0.002	0.03573
xAG	pre-COVID-19	1500	0.161	0.25664				
	post-COVID-19	1500	0.1279	0.21529	3.831	2909.982	0	0.03313
SCA	pre-COVID-19	1500	2.7953	2.44679				
	post-COVID-19	1500	2.4207	2.33023	4.295	2998	0	0.37467
LongCmp	pre-COVID-19	1500	1.5253	1.8711				
	post-COVID-19	1500	1.3527	1.68944	2.653	2998	0.008	0.17267
Touches	pre-COVID-19	1500	35.1313	18.94677				
	post-COVID-19	1500	33.25	19.74536	2.663	2998	0.008	1.88133
Mid3rd	pre-COVID-19	1500	14.9613	9.47887				
	post-COVID-19	1500	14.1947	9.78574	2.179	2998	0.029	0.76667
Att3rd	pre-COVID-19	1500	17.556	11.20112				
	post-COVID-19	1500	16.4987	11.3053	2.573	2998	0.01	1.05733
AttPen	pre-COVID-19	1500	3.5767	2.88958				
	post-COVID-19	1500	3.236	2.90297	3.221	2998	0.001	0.34067
TouchesLive	pre-COVID-19	1500	35.0807	18.92895				
	post-COVID-19	1500	33.2087	19.72063	2.652	2992.981	0.008	1.87200
DriAtt	pre-COVID-19	1500	2.2447	2.32014				
	post-COVID-19	1500	2.0687	2.31103	2.082	2998	0.037	0.17600
DriSucc	pre-COVID-19	1500	1.2693	1.58865				
	post-COVID-19	1500	1.1147	1.48245	2.757	2983.761	0.006	0.15467
DriSucc%	pre-COVID-19	1500	41.5604	39.85161				
	post-COVID-19	1500	37.1386	39.16006	3.065	2998	0.002	4.42180
ReceivingRec	pre-COVID-19	1500	26.874	15.71675				
	post-COVID-19	1500	25.3467	16.11127	2.628	2998	0.009	1.52733

Differences in MTP indicators between pre- and post-COVID-19 infection are displayed as median differences with 95% confidence intervals.

Independent-samples t test was conducted to compare the MTP indicators of the elite group and the control group before and after contracting COVID-19 (Table 4). The results showed that thirteen indicators in the control group exhibited significant differences (*p* < 0.05) before and after infection, while only seven indicators in the elite group showed significant differences. Among the same six indicators significantly affected after contracting COVID-19 in both group, the control group demonstrated a greater decrease in xG, Att Pen, and DriSucc% compared to their counterparts in the elite group. On the other hand, indicators such as npxG, xAG, and SCA showed a smaller negative impact on the control group after contracting COVID-19 than on the elite group.

**Table 4:** Comparison of MTP indicators of the elite and control groups after COVID-19 infection.

Elite group									Control group								
Indicators significantly affected after COVID-19 infection in both the elite group and the control group																	
Indicators	Group	N	Mean	SD	t	df	Two-sided p	Mean difference	Indicators	Group	N	Mean	SD	t	df	Two-sided p	Mean difference
xG	pre-COVID-19	750	0.3832	0.43824					xG	pre-COVID-19	750	0.2215	0.30119				
	post-COVID-19	750	0.3341	0.41566	2.225	1493.827	0.026	0.04907		post-COVID-19	750	0.1721	0.26922	3.344	1479.531	0.001	0.04933
npxG	pre-COVID-19	750	0.3299	0.37472					npxG	pre-COVID-19	750	0.1861	0.26006				
	post-COVID-19	750	0.2873	0.34612	2.283	1488.656	0.023	0.04253		post-COVID-19	750	0.1572	0.24209	2.23	1490.383	0.026	0.02893
xAG	pre-COVID-19	750	0.2155	0.30153					xAG	pre-COVID-19	750	0.1065	0.18695				
	post-COVID-19	750	0.1700	0.24689	3.195	1441.864	0.001	0.04547		post-COVID-19	750	0.0857	0.16807	2.266	1481.335	0.024	0.02080
SCA	pre-COVID-19	750	3.3707	2.67085					SCA	pre-COVID-19	750	2.2200	2.04577				
	post-COVID-19	750	2.9773	2.56312	2.91	1495.468	0.004	0.39333		post-COVID-19	750	1.8640	1.91757	3.477	1491.77	0.001	0.35600
AttPen	pre-COVID-19	750	4.4773	3.1566					AttPen	pre-COVID-19	750	2.6760	2.26315				
	post-COVID-19	750	4.1533	3.20284	1.973	1498	0.049	0.32400		post-COVID-19	750	2.3187	2.21864	3.088	1497.409	0.002	0.35733
DriSucc%	pre-COVID-19	750	42.5921	38.72237					DriSucc%	pre-COVID-19	750	40.5287	40.94958				
	post-COVID-19	750	38.8067	38.00334	1.911	1498	0.056	3.78547		post-COVID-19	750	35.4705	40.23981	2.413	1497.542	0.016	5.05813
Indicators that are affected after COVID-19 infection in each group																	
LongCmp	pre-COVID-19	750	1.8000	1.99465	2.145	1498	0.032	0.21200	GCA	pre-COVID-19	750	0.2773	0.54969				
	post-COVID-19	750	1.5880	1.82977						post-COVID-19	750	0.2280	0.51934	1.787	1493.193	0.074	0.04933
LongCmp%	pre-COVID-19	750	48.5707	38.49787	2.258	1498	0.024	4.49067	ShortCmp	pre-COVID-19	750	8.9080	6.33665				
	post-COVID-19	750	44.0800	38.5166						post-COVID-19	750	8.3547	6.62476	1.653	1495.048	0.099	0.55333
									Touches	pre-COVID-19	750	29.8440	15.8979				
										post-COVID-19	750	27.3080	17.47154	2.94	1484.85	0.003	2.53600
									Def3rd	pre-COVID-19	750	3.1493	3.29082				
										post-COVID-19	750	2.8347	3.26643	1.859	1497.917	0.063	0.31467
									Mid3rd	pre-COVID-19	750	13.3213	8.30699				
										post-COVID-19	750	12.4507	9.29502	1.913	1479.471	0.056	0.87067
									Att3rd	pre-COVID-19	750	13.8587	8.64591				
										post-COVID-19	750	12.3933	8.74249	3.264	1497.815	0.001	1.46533
									TouchesLive	pre-COVID-19	750	29.8053	15.88618				
										post-COVID-19	750	27.2893	17.46292	2.919	1484.783	0.004	2.51600
									DriAtt	pre-COVID-19	750	1.9587	2.04741				
										post-COVID-19	750	1.7293	1.98662	2.202	1496.641	0.028	0.22933
									DriSucc	pre-COVID-19	750	1.1387	1.43557				
										post-COVID-19	750	0.9693	1.34368	2.358	1491.492	0.018	0.16933
									DriMis	pre-COVID-19	750	1.8893	1.62582				
										post-COVID-19	750	1.7267	1.74433	1.868	1490.645	0.062	0.16267
									ReceivingRec	pre-COVID-19	750	22.0613	12.51078				
										post-COVID-19	750	20.3347	13.47544	2.572	1489.811	0.01	1.72667
									ReceivingProg	pre-COVID-19	750	4.2547	3.11985				
										post-COVID-19	750	3.9213	3.20971	2.039	1496.794	0.042	0.33333

Differences in MTP indicators between pre- and post-COVID-19 infection are displayed as median differences with 95% confidence intervals.

The control group’s players were more impacted in the possession category, particularly in Touches, Take-ons, and Receiving. Notably, the control group's attackers had 2.536 fewer touches per game, 1.465 fewer receptions per game in Att 3rd, and 1.727 fewer receptions per game in ReceivingRec after contracting COVID-19. In contrast, the elite group's attackers exhibited a significant decline in long-passing ability, with a 4.491% reduction in success rate.

Additionally, certain indicators exhibited marginal significance differences (0.1 > *p* > 0.05), such as DriSucc% in the elite group and GCA, ShortCmp, Def 3rd, Mid 3rd and DriMis in the control group. While the elite group’s success rate in dribbling marginally decreased after COVID-19 infection, the 3.785% reduction per game was considerably lower than the control group’s 5.058% reduction. These findings supported Hypothesis H1b.

### Average 3.09 matches for MTP of attacking players to recover to pre-COVID-19 levels; the elite group recovers more quickly than the control group

**Figure 4:**
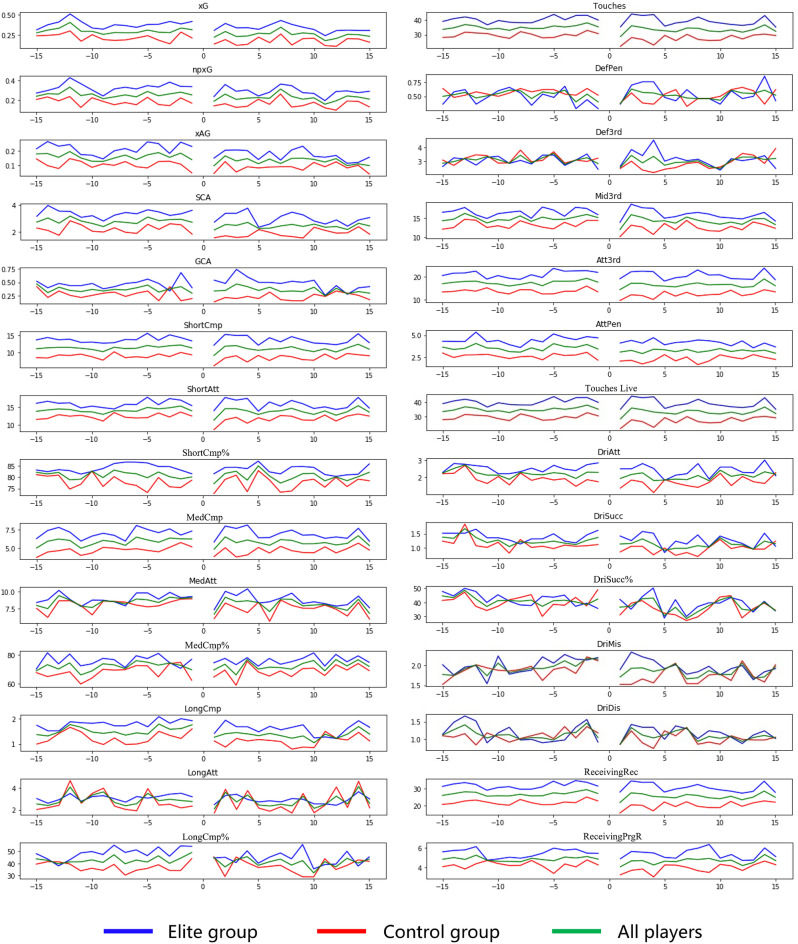
Changes in players' MTP indicators over 15 matches before and after COVID-19 infection. In all subplots, the horizontal axis represents the monitored games. The notation − 15 corresponds to the 15th game before COVID-19 infection, while 15 signifies the 15th game after COVID-19 infection, and so forth. The vertical axis of all subplots illustrates the data for various indicators.

Figure 4 reveals that several indicators experienced a notable decline in players’ first match after COVID-19 infection but gradually improved in subsequent matches. On average, it took 3.09 matches for the MTP indicators of 100 attacking players to return to their pre-COVID-19 levels. The indicators in the scoring, passing, and possession categories required 3.65, 2.88, and 3.03 matches to recover to the level before the players contracted COVID-19, respectively. Among them, ShortCmp% needed the fewest matches at 1.83, while GCA required the most matches at 4.21.

**Figure 5:**
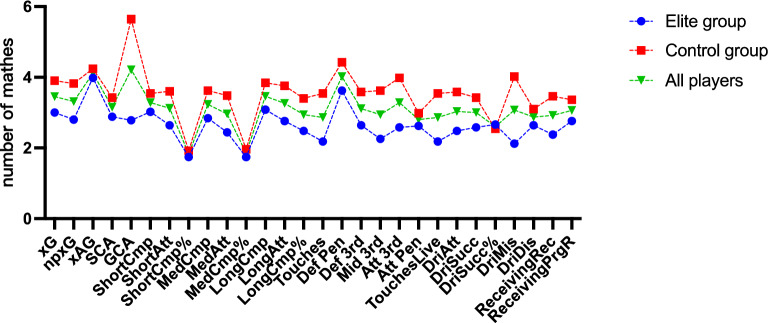
Average matches needed for players' indicators to recover to pre-COVID-19 levels after infection.

The control group players, on average, needed 3.55 matches to recover their MTP indicators to pre-COVID-19 levels, which was higher than the elite group’s average of 2.64 matches (Fig. 5). The most substantial difference was observed in GCA, where the elite group needed 2.78 matches compared to the control group’s 5.64 matches. Notably, only the DriSucc% indicator had a lower average number of matches for the control group (2.54 matches) compared to the elite group (2.66 matches). Overall, the majority of MTP indicators for the elite group players needed fewer matches to return to their pre-COVID-19 levels than the control group, indicating a quicker recovery for the elite group players. Hypothesis 2 was partially validated.

### Age did not have a significant influence on the recovery of MTP for attacking players

We conducted linear regression analysis using age as the independent variable and xG, npxG, and other MTP indicators as dependent variables to investigate whether the number of matches needed for attacking players to return to their pre-COVID-19 performance level significantly increased with age. No indicator supported this hypothesis in the study. This remained consistent when separately testing the elite and control groups. Consequently, attacking players recovering from COVID-19 and returning to matches do not experience a significant slowdown in the recovery of various MTP indicators due to older age. Hypotheses H3a and H3b were not supported.

### Limited impact of age on changes in MTP indicators for attacking players after contracting COVID-19

We hypothesize that the players’ MTP may decrease with age. Furthermore, we conjecture that after contracting COVID-19, the extent to which players’ MTP decreases due to aging is greater than the extent before contracting COVID-19.

**Figure 6:**
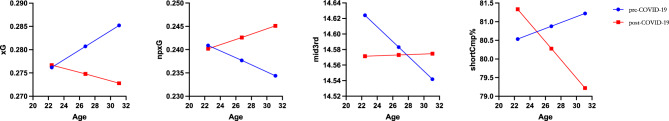
Age-related changes in xG, npxG, Mid3rd, and ShortCmp% before and after COVID-19 infection.

As depicted in Fig. 6, a comprehensive analysis of all 100 attacking players indicates that only xG, ShortCmp%, and Mid3rd were significantly influenced when the player contracted COVID-19. xG significantly increased with age before contracting COVID-19 (*p* = 0.019), but this association lost significance after infection (*p* = 0.307). Mid3rd exhibited a significant decrease with age before COVID-19 infection (*p* = 0.021), and npxG showed a marginally significant decrease with age before COVID-19 infection (*p* = 0.077). However, these relationships lost significance after COVID-19 infection. ShortCmp% significantly decreased with age after COVID-19 infection (*p* = 0.015), whereas no significant relationship was observed between age and this variable before COVID-19 infection. Thus, among all the indicators, only ShortCmp% provided support for hypothesis H4a. The analysis demonstrated that for each unit increase in age, ShortCmp% decreased by 0.244 units after COVID-19 infection (effect = − 0.244, *p* = 0.015, LLCI = − 0.441, ULCI = − 0.047).

**Figure 7:**
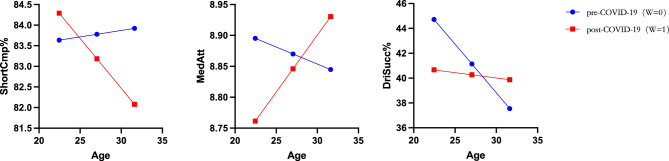
Age-related changes in ShortCmp%, MedAtt, and DriSucc% before and after COVID-19 infection in the elite group.

In the elite group, ShortCmp% significantly decreased with age after COVID-19 infection (*p* = 0.015), and there was a marginal increase in MedAtt associated with aging (*p* = 0.064). However, these two indicators exhibited no significant changes before players contracted COVID-19. Pre-COVID-19 infection, DriSucc% significantly decreased with age (*p* = 0.001), but no significant change occurred after COVID-19 infection (Fig. 7).

**Figure 8:**
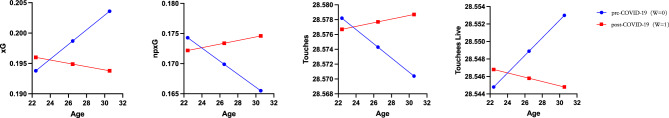
Age-related changes in xG, npxG, Touches, and Touches Live before and after COVID-19 infection in the control group.

Before contracting COVID-19, in the control group, xG (*p* = 0.002) and Touches Live (*p* = 0.034) increased with age, while npxG (*p* = 0.005) and Touches (*p* = 0.045) decreased with age. However, after COVID-19 infection, none of these indicators exhibited significant age-related changes (Fig. 8).

Overall, only the ShortCmp% indicator in the elite group supported hypothesis H4b. The analysis demonstrated that with each unit increase in player age, there was a corresponding decrease of 0.242 units (effect = − 0.242, *p* = 0.015, LLCI = − 0.436, ULCI = − 0.047) in this specific indicator after contracting COVID-19.

## Discussion

In a football match, what directly impacts match results are actions involving the ball, including possession, passing, and shooting^78^. These collectively constitute a player’s MTP.

In practice, distinguishing whether the COVID-19 virus itself directly affects a player’s MTP or if it is the prolonged period without systematic training and matches after contracting COVID-19 that induces changes in a player’s MTP can be challenging. Researchers find that longer mid-season may negatively affect momentum and performance^55^. The emergence of the COVID-19 pandemic has exacerbated this situation. However, for this study, we do not need to make this differentiation. We treat these aspects as a comprehensive and inseparable factual scenario. Our primary focus lies in assessing whether significant differences exist in various MTP indicators between players who have resumed play after contracting COVID-19 and their corresponding indicators before infection.

Notably, a substantial majority of players who contracted the virus, amounting to 76%, demonstrated significant alterations in at least one MTP indicator upon their return to matches. After returning to the field, attacking players recovering from COVID-19 exhibited notable differences in fourteen MTP indicators, including xG and npxG, in comparison to their pre-COVID-19 levels. These indicators fall within the scoring, passing, and possession categories. Our study reveals a significant decrease in all five scoring-related MTP indicators (xG, npxG, xAG, SCA, and GCA) among attacking players post-COVID-19 infection. This indicates a clear negative impact on attacking players post-COVID-19, given that scoring goals and assisting teammates is their primary match task^79,80^.

In the possession category, players exhibited the most indicators with significant decreases in matches post-COVID-19. Furthermore, players also experienced a decrease in receiving subcategories. This phenomenon can be interpreted from multiple perspectives. From a team-wide viewpoint, the reduction in team training due to COVID-19 restrictions left players tactically underprepared. The lack of training time and collective tactical work may have led teams to play a more compact game to avoid potentially costly mistakes^81^. From an individual player's perspective, these findings suggest that attacking players exhibit a reduced desire or ability to attack in the initial 15 matches after COVID-19 recovery. This decline in attacking desire or ability may stem from the impact of COVID-19 on their physical function and the absence of systematic training and matches, leading to their physical fitness not fully returning to optimal levels. Studies find that players may face challenges in positioning themselves correctly and executing appropriate movements due to compromised physical performance (e.g., endurance and speed), resulting in difficulties in carrying out their attacking tasks^47,48^.

In our study, six out of seven indicators in the elite group coincide with the indicators exhibiting a substantial decline in the control group after resuming play post-COVID-19 infection. Furthermore, the control group has seven additional indicators where the elite group did not show a notable decline upon returning to matches. This observation emphasizes that, although the control group has a lower average number of indicators displaying significant differences after contracting COVID-19 compared to the elite group, the absolute count of affected indicators remains relatively high, nearly doubling that of the elite group. This finding is similar to observations in other sports, where a wide range of examples suggests that several athletic performance indices, such as acceleration, endurance, or reaction rate, do not decline, despite previous reports of adverse effects of SARS-CoV-2 infections on maximal aerobic capacity and the nervous system in populations of nonelite athletes^82,83^. This could be related to top players having better physical conditions^84^ and usually being under strict medical control^85^.

Numerous studies support the notion that players tend to experience a decline in skill performance when they have poor physical fitness or undergo significant physical exhaustion, such as in the second half of a match^54,86,87^. The substantial reduction in the number of attempted long passes by players after COVID-19 recovery further corroborates this perspective. Executing long passes imposes greater demands on players’ leg strength and other physical fitness aspects compared to short and medium-range passes. This aligns with previous research indicating a decrease in physical fitness indicators among players from La Liga, Serie A, Croatian HNL, and Polish Ekstraklasa after contracting COVID-19^37^.

For the same indicators significantly affected after contracting COVID-19 in both groups, the control group exhibited a more substantial decrease in xG, Att Pen, and DriSucc% compared to the elite group. Conversely, the indicators npxG, xAG, and SCA, which all fall within the scoring category, showed a less pronounced decrease in the control group after COVID-19 infection compared to the elite group. However, upon comparing the mean values of these indicators between the two groups, it was evident that the control group's attackers had significantly lower data than the elite group both before and after contracting COVID-19. We posit that the control group's attackers having inherently lower MTP data contributed to the limited reduction in their MTP data even after contracting COVID-19. Consequently, their reduction in the mentioned MTP indicators after contracting COVID-19 was smaller than that of the elite group. This observation is consistent with the notion that top players bear the primary responsibility for scoring within their teams^80^.

In the control group, indicators significantly affected after contracting COVID-19 were primarily concentrated in the possession category, specifically in the subcategories of Touches, Take-ons, and Receiving. This implies that the technical skills of the control group players suffered more adverse effects after contracting COVID-19 compared to the elite group. Although the elite group’s success rate in dribbling exhibited a marginally significant decrease after contracting COVID-19 compared to before, the decrease of 3.79% per match was considerably lower than the control group's decrease of 5.06% per match. This discovery once again confirms that, in practical terms of matches, the negative impact on elite players is smaller than that on nonelite athletes^82,83^.

Shots on target appeared as a strong indicator to be considered and maximized by football teams, being more relevant than ball possession and passing for success^78^. Considering that scoring is the primary task for an attacking player, followed by possession and passing^79^, the difficulty of completing these tasks should also follow that order. This is because, in matches, defenders of opponents are likely to prioritize limiting the scoring ability of attacking players, followed by their possession and passing abilities^88^. The recovery speed of MTP-related indicators in matches after players contracted COVID-19 supports this perspective. On average, the MTP indicators for scoring, possession, and passing require 3.65 matches, 3.03 matches, and 2.88 matches, respectively, to return to the pre-COVID-19 level. Among all the indicators, ShortCmp% requires only 1.83 matches, while GCA requires the most, with 4.21 matches. The recovery of players’ short passing ability is the quickest, which is related to players opting to play a more compact game to avoid potentially costly mistakes after returning to the field^81^. It may also be related to player behavior being influenced by tactical demands and/or conscious or unconscious self-protection^11^. After all, short passing is one of the most fundamental techniques in football and one of the safest to perform^89^. Conversely, GCA, which represents a player's offensive actions directly leading to a goal, such as passes, take-ons, and drawing fouls, is a core indicator in the scoring category^90^. It demands much more from players than short passing, making it relatively the hardest to recover.

Elite group players' MTP indicators require an average of 2.64 matches to return to the pre-COVID-19 level, whereas the control group requires 3.55 matches for recovery. The most substantial difference between the two groups is observed in GCA, with the elite group requiring 2.78 matches for recovery, whereas the control group needs 5.64 matches. Our findings indicate that the GCA of control group players before contracting COVID-19 was 0.28, whereas for the elite group, it was 0.47. Referring to the earlier discussion on the significance of GCA in evaluating attacking players, it can be concluded that there is a significant difference between the control group and the elite group, both from the MTP indicator data and the recovery situation. Given that the most important tasks for an attacker are scoring and assisting^79^, this finding illustrates that the greatest advantage of the elite group over the control group lies precisely in this aspect, which also reflects the value of elite group players.

A study on athletes’ recovery after contracting COVID-19 indicated that advancing age was linked to a more extended recovery period following COVID-19. Additionally, the prolonged impact of COVID-19 on the recovery to engage in sports seems more protracted in athletes over thirty^91^. Nevertheless, no significant difference is observed based on age when evaluating the number of matches needed for all 100 attacking players to restore their MTP indicators to the pre-COVID level.

When exploring whether age’s impact on MTP indicators is moderated by COVID-19 infection, we observed similar results. Regardless of whether players contracted COVID-19, age exerted limited influence on MTP indicators. Specifically, only ShortCmp% exhibited a significant decrease with increasing age after COVID-19 infection. This observation aligns with the conclusions drawn from the separate analysis of the elite group but does not extend to the control group. The aforementioned research conclusion may be related to the fact that all the players in the sample come from the Big Five. The Big Five boasts the world’s finest football players and professional management team^68,69^, so in this regard, even the control group’s players did not show a significant difference from the elite group's players.

Our study offers a different perspective from Fischer et al.’ s research, which observed a more substantial decline in pass performance among players over 30 compared to those under 25 and those between 26 and 30^57^. It's worth noting that their study focused exclusively on Bundesliga and Serie A players, excluding representation from the Premier League, La Liga, and Ligue 1, potentially limiting the generalizability of their findings to a broader range of elite football players all over the world. Additionally, their inclusion of players from all positions differs from our research, which specifically concentrates on attacking players. Furthermore, the two-season span of their study (2019/2020 and the 2020/2021 seasons) may present challenges in capturing the nuanced impact of age on performance over a more extended period. In contrast, our study aligns with the findings of Rey et al*.*, whose longitudinal study exploring the impacts of aging on match performance in elite football revealed that, despite players with extensive careers struggling to maintain match-related physical performance as they age, they can offset these declines by annually enhancing their technical and tactical skills with increasing age^62^.

## Conclusion

### Recovery and performance insights

This longitudinal study illuminates the match technical performance (MTP) of football attacking players recovering from COVID-19 upon their return to the field. Despite experiencing some negative impact on their MTP after contracting COVID-19, these players demonstrate a relatively swift recovery to their previous level upon resuming matches. Notably, top-level attacking players from the Big Five European Leagues, with their extensive experience and superior overall abilities, exhibit less vulnerability to the negative impact and showcase faster recovery compared to their average-level counterparts. Moreover, age does not significantly impede the MTP of attacking players recovering from COVID-19 upon their return to the field.

### Practical implications

Implementing these findings can assist football coaches and managers in developing more targeted recovery training programs for players post-COVID-19 infection or related illnesses. There should be a focus on technical drills aimed at restoring skills that take longer to recover and those most affected in terms of MTP upon returning to play. This study's insights into the impact on MTP and the recovery timeline provide strategic guidance for match preparation, particularly in setting specific tasks and objectives for players in their first few matches back on the field.

This research was exclusively dedicated to attackers within the Big Five. Future studies could extend to include players from various league levels, incorporate more positions, and also cover youth and women’s football, broadening the scope and applicability of the findings.

## Data Availability

The datasets generated during and/or analyzed during the current study are available on the official websites of each of the Big Five leagues, as well as on FBref.com, [https://fbref.com]. Further queries can be directed to the authors.
